# Bleeding management in adolescent idiopathic scoliosis: the role of low-dose tranexamic acid

**DOI:** 10.1016/j.bjane.2026.844746

**Published:** 2026-03-07

**Authors:** Paula Alexandra Sá, Filipa Pereira, Daniel Soares, António Oliveira, Eugénia Cruz, Sibylle Langenecker

**Affiliations:** a Centro Hospitalar Universitário de Santo António, Unidade Local de Saúde de Santo António, Department of Anesthesiology, Intensive Care and Emergency, Porto, Portugal; b Centro Hospitalar Tâmega e Sousa, Department of Anesthesiology, Intensive Care and Emergency, Penafiel, Portugal; c Centro Hospitalar Universitário de Santo António, Unidade Local de Saúde de Santo António, Department of Ortopedics, Porto, Portugal; d Centro Hospitalar Universitário de Santo António, Unidade Local de Saúde de Santo António, Department Imuno-Hemotherapy, Porto, Portugal; e Universidade do Porto, Instituto Ciências Biomédicas Abel Salazar, Porto, Portugal; f Evangelical Hospital Vienna and Sigmund Freud Private University, Department of Anesthesia and Intensive Care, Vienna, Austria; g Sigmund Freud Private University, Campus Prater, Vienna

**Keywords:** Adolescent, Blood transfusion, Hemorrhage, Scoliosis, Spinal fusion, Tranexamic acid

## Abstract

**Background:**

Despite advances in surgical and blood management techniques, it continues to carry risks of excessive blood loss and transfusion. Tranexamic Acid (TXA), an antifibrinolytic agent, has shown efficacy in reducing these risks across various surgeries. This retrospective cohort study evaluated factors influencing intraoperative blood loss in Adolescent Idiopathic Scoliosis (AIS) surgery, focusing on low-dose TXA administration.

**Methods:**

This retrospective cohort study included 187 AIS patients undergoing posterior spinal fusion with or without intraoperative TXA. Patients were grouped into non-TXA (116 patients) and TXA (71 patients) cohorts. The TXA group received a 10 mg.kg^-1^ intravenous loading dose over 15 minutes, followed by a continuous infusion of 1 mg.kg^-1^.h^-1^ until skin closure. Outcomes included estimated blood loss, transfusion needs, length of stay, and thromboembolic or neurologic complications. Multivariate regression adjusted intraoperative blood volume loss for potential covariates.

**Results:**

Baseline demographic characteristics were similar between groups. However, differences in surgical complexity cannot be excluded and are acknowledged. TXA use was associated with a 39% reduction in estimated intraoperative blood volume loss compared with the non-TXA group. Blood loss correlated significantly with TXA use, sex, ASA status, number of fused levels, curve type, and surgery duration.

**Conclusion:**

Low-dose TXA significantly reduced intraoperative blood loss and transfusion requirements during AIS surgery. Greater blood loss was linked to longer procedures and more fused levels, whereas lumbar curve type appeared protective. This study provides insights into AIS outcomes and their associations with predictive factors and TXA use.

**Level of evidence:**

Level III – retrospective cohort study.

## Introduction

Scoliosis is a three-dimensional deformity of the spine, including a lateral deviation of the normal vertical line equal to or greater than 10 degrees, rotation of the vertebral body, and a paravertebral hump. Primary curves are the earliest to appear and occur most frequently in the thoracic and lumbar regions.^1^ The magnitude of scoliosis severity is mainly measured using the Cobb method. Adolescent Idiopathic Scoliosis (AIS) is the most common form of scoliosis, affecting 1%‒4% of adolescents.^1^ Surgical treatment involves extensive soft tissue dissection and significant bone bleeding during instrumentation and decortications, there for transfusions of blood may possible necessary. Meantime, there is strong evidence that transfusion-related side effects are associated with increased morbidity and mortality, such as transfusion-related infections, transfusion-related acute lung injury, transfusion-related acute circulatory overload, and transfusion-related immunomodulation.^2^ Therefore, decreasing blood loss and transfusion requirements should improve patients’ safety, their post-operative recovery, and their long-term outcome.^2^^,^^3^ Factors related to the individual patient that influence blood loss during surgery include gender, height, weight, severity and type of spinal deformity, as well as surgical factors like operative time, the procedure performed, surgical approach, number of vertebrae fused, number of anchors (pedicle screws, hooks, and wires) placed, average mean arterial pressure during the procedure, blood salvage techniques and the use of antifibrinolytic medications.^4^ Therefore, the etiology of blood loss is multifactorial. However, activation of fibrinolysis plays a central role in bleeding during scoliosis surgery. Therefore, antifibrinolytic drugs can serve as a valuable resource in this context.

The concepts of a ceiling effect and dose-dependent side effects for Tranexamic Acid (TXA) are well documented, particularly neurological complications such as seizures, which increase with higher doses of tranexamic acid. High-dose regimens have also been associated with a higher incidence of thromboembolic events and other adverse effects without additional efficacy. Nevertheless, TXA has been used in this population due to the growing evidence supporting its safety profile.^5^ TXA is a synthetic derivative of the amino acid lysine that exerts its antifibrinolytic effect through the reversible blockade of lysine binding sites on plasminogen molecules, reducing fibrin degradation.^5^ Chen et al., in a meta-analysis, found that TXA treatment significantly reduces blood transfusion demand and blood loss, but the results should be interpreted with caution due to high heterogeneity and limited data.^6^ Our retrospective observational cohort study uses a homogenized patient population, and all surgeries are performed by the same team of surgeons and anesthesiologists, which can substantially reduce clinical and methodological variability. This approach directly addresses key sources of heterogeneity identified in meta-analyses and systematic reviews, such as differences in patient baseline characteristics, surgical technique, and perioperative management, which have been shown to influence outcomes like blood loss, transfusion rates, and adverse events with tranexamic acid.^7^, ^8^, ^9^ By minimizing these variables, our study can provide more precise estimates of the effectiveness and safety of tranexamic acid and help clarify whether observed heterogeneity in pooled analyses is due to true differences in intervention effect or confounding factors.

This study aimed to evaluate the association between low-dose TXA use and percentage of intraoperative estimated blood volume loss in patients undergoing surgery for adolescent idiopathic scoliosis, adjusted for known confounders, to better characterize TXA use in this population.

## Methods

A retrospective cohort study was performed in a university hospital in Portugal, approved by Ethical Research Committee on June 5, 2015, with the registration number 2015.083 (077-DEFI/072-CES). We reviewed medical records and operative reports of surgically treated patients during five consecutive years.

During the study period, this retrospective cohort comprised two distinct stages: a pre–September 2012 stage, during which TXA was not administered, and a post-implementation stage following the introduction of TXA, during which was administered to all patients unless. unless contraindicated.

### Participants and procedures

We included patients under 18 years who underwent PSF with pedicle screw or hybrid hook-screw constructs and had complete medical records. Patients with scoliosis of other etiologies, revision surgery, or combined posterior-anterior fusion were excluded. Patients’ data collected included age, gender, body weight, ASA status, curve angle, anchors used, levels fused, surgery duration, and hospital stay. Intraoperative blood loss and transfusion data were obtained from operative reports and chart reviews.

Anesthetic management was standardized using intravenous total anesthesia with Target Controlled Infusion of sufentanil (PK model Gepts et al.)^10^ and propofol (PK model Schnider et al.).^11^ Rocuronium was given for intubation. Anesthesiologists maintained mean arterial pressure (MAP) of 60 mmHg during exposure/anchor placement and 70–90 mmHg during correction. Monitoring included pulse oximetry, ECG, invasive pressure, neuromuscular blockade (TOF), and core temperature. Normothermia was maintained with forced-air warming and fluid warmer.

Intraoperative somatosensory and motor evoked potential monitoring was performed in all patients. Fluid therapy, blood loss, and transfusion were guided by arterial pressure, hematocrit, and blood gas measurements. No predefined hemoglobin trigger for transfusion existed; decisions relied on clinical judgment. When indicated, advanced hemodynamic monitoring with PulsionFlex® (ProAQT) was added. Surgical technique was consistent across cases and performed by senior orthopedic surgeons.

### TXA treatment group

TXA group: an intravenous infusion of 10 mg.kg^-1^ of TXA was administered over 15 minutes, 15 minutes before surgical incision, followed by perfusion of 1 mg.kg^-1^.h^-1^ since incision up to surgical wound closure after surgery.

### Outcome parameters

The primary outcome was the percentage of intraoperative estimated blood volume loss. Blood loss was quantified by weighing all surgical swabs using a calibrated analytical scale and measuring the volume of blood in the suction canister after subtracting irrigation fluids. Total blood loss was then expressed as a percentage of each patient’s estimated blood volume, thereby standardizing blood loss relative to patient size.

This was done using the formula: Estimated Blood Loss (EBL) / Estimated Blood Volume (EBV) × 100. The EBV was determined to be 70 mL.kg^-1^ of body weight.^12^ This approach offers a more physiological indication of the extent of blood loss for each patient. The number of units of Packed Red Blood Cells (PRBCs) transfused intraoperatively and during the postoperative course was recorded and analyzed primarily as a binary outcome (i.e., whether a patient received a transfusion or not), rather than by the total volume of blood transfused.

During hospitalization post-surgery, all patients were monitored for clinical signs of complications, including venous thromboembolic events and seizures. Surgeons assessed these patients in outpatient clinics at intervals of two weeks, three months, and one-year post-surgery. The length of stay was assessed for each patient. Fused levels were calculated by counting all levels from the proximal level to the distal level.

### Statistical analysis

Categorical variables were described using absolute and relative frequencies (%). Numeric variables were summarized as means with Standard Deviations (SD) for normally distributed data, and as medians with interquartile ranges for non-normal distributed data. Normality was verified using the Shapiro-Wilks test and histograms. Group differences were analyzed with Student’s *t-*test or Mann-Whitney *U*-test, and proportions with Chi-square or Fisher’s exact test. Associations between variables were explored with Pearson’s or Spearman’s coefficients. Missing data were assessed for all variables and handled using a complete-case analysis. Patients with missing information on the outcome (percentage of intraoperative estimated blood volume loss), exposure (TXA), or key covariates were excluded, resulting in 9.7% of patients being excluded from the primary analysis. This approach ensured a consistent analytical sample across all variables, and the characteristics of excluded patients were reported to assess potential selection bias.^13^

Univariable and multivariable linear regression models were used to estimate the association between TXA use and blood loss expressed as a percentage of estimated blood volume, adjusting for prespecified clinically relevant covariates (age, sex, weight, ASA status, Cobb angle, number of levels, number of pedicle screws, and duration of surgery). Because TXA exposure was determined by study period, calendar year and era were not included in the primary adjusted model, as they were inherent to the exposure definition. For the multivariable regression models, covariates were selected a priori based on clinical relevance and not only on univariable statistical significance. The multivariable regression model included prespecified clinically relevant covariates and was evaluated for linearity, homoscedasticity, and normality of residuals (Fig. S7 in the Supplementary Material). Linearity and variance patterns were examined using residual-versus-fitted plots, normality was assessed with quantile-quantile and cumulative distribution plots, and heteroscedasticity was tested using the Breusch-Pagan/Cook-Weisberg test. Because heteroscedasticity was present, models were fitted using heteroscedasticity-consistent (robust) standard errors. Regression coefficients with 95% Confidence Intervals were reported. Several sensitivity analyses were performed to evaluate the robustness of model specification and potential confounding. First, for specification sensitivity models examined the alternative inclusion of surgical variables (number of fused levels and number of pedicle screws) given observed baseline imbalances. Three models were fitted, excluding (i) Screws only, (ii) Fused levels only, and (iii) Both screws and fused levels, while retaining all other covariates.

Second, a propensity score sensitivity analysis was conducted to address potential confounding by indication. A propensity score for receiving TXA was estimated using logistic regression, including age, weight, gender, ASA status, Cobb angle, number of fused levels, number of screws, duration of surgery, and scoliosis type. Baseline covariate balance before weighting was assessed using Standardized Mean Differences (SMDs), with values < 0.10 indicating adequate balance. Stabilized Inverse Probability of Treatment Weights (IPTW) were then calculated to create a weighted pseudo-population balanced on measured covariates. Two weighted linear regression models were fitted: (i) A marginal IPTW model including TXA only and (ii) A doubly robust IPTW model additionally adjusting for the covariates included in the multivariable model. Propensity-score overlap, and weight distributions were examined graphically. Data were analyzed using Stata 17.0. We used the STROBE reporting guideline to draft this manuscript, included in Table S1 in the Supplementary Material.^14^

## Results

### Participants and procedures

In total, 207 patients’ perioperative records were evaluated from January 2009 to December 2014; 187 met the inclusion criteria Fig. S1 in the Supplementary Material (information about blood loss was missing in 16 patients, data regarding TXA administration were not available in 2 patients, and the Cobb angle was not registered in 1 patient, one was a kyphosis correction). Patients' demographics are presented for both the TXA and non-TXA groups in Table 1. One hundred sixteen (106 female and 10 males; mean age 14.6 years) did not receive TXA, while 71 did (62 females and 9 males; mean age 14.7 years). The frequency of female patients was higher in both groups (comparably so), which is an epidemiological characteristic of this population that has been well described in the literature.^15^

**Table 1:** Demographic and preoperative data of adolescents who underwent surgery for idiopathic scoliosis with or without Tranexamic acid (TXA).

		Non TXA (n = 116)		TXA (n = 71)		*p*-value
**Age (years)^a^**		14.6	(1.8)	14.7	(1.8)	0.659^1^
**Sex, n (%)**	Female	106	(91%)	62	(87%)	0.373^2^
	Male	10	(9%)	9	(13%)	
**Weight (kg)^b^**		53	(46‒59)	53	(49‒58)	0.806^3^
**ASA class, n (%)**	I	62	(53%)	37	(52%)	0.201^4^
	II	49	(42%)	34	(48%)	
	III	5	(4%)	0	(0%)	
**Type of curve, n (%)**	Dorsal	83	(72%)	52	(73%)	0.834^4^
	Lumbar	15	(13%)	7	(10%)	
	Dorsolumbar	18	(15%)	12	(17%)	
**Cobb angle (^b^)**		58.6	(48.7‒67.9)	54.2	(46‒68)	0.304^3^

Values are presented as the ^a^ mean (SD), ^b^ median (Q1‒Q3), or number (%).

^1^ t-test; ^2^ χ2 test; ^3^ Mann-Whitney test; ^4^ Fisher test.

ASA, American Society of Anesthesiologists.

**Figure. 1:**
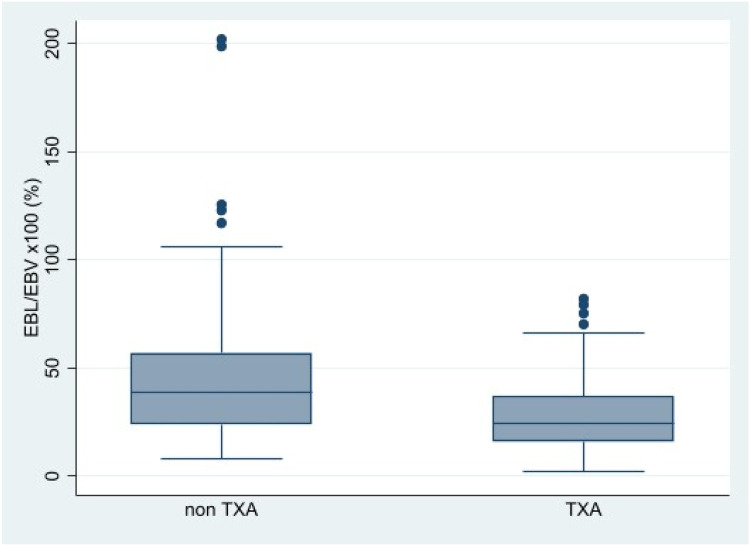
Box plot representing intraoperative blood loss (mL) in the non-Tranexamic Acid (TXA) group versus the TXA group. Outliers are represented as data dots 1.5 times the interquartile range.

Intraoperative and postoperative variables are shown in Table 2. There were significant differences between the two groups for the number of anchors (*p* < 0.001) and duration of surgery (*p* = 0.008). Blood loss and transfusion data are presented in Table 3. SMDs confirmed moderate baseline imbalances in these covariates (Table S2 in the Supplementary Material). The percentage of the patient´s estimated blood volume loss was approximately 39% lower in the TXA group compared to the non-TXA group (Fig. 1).

**Table 2:** Intraoperative and postoperative data of adolescents who underwent surgery for idiopathic scoliosis with or without Tranexamic acid (TXA).

	Non TXA (n = 116)		TXA (n = 71)		*p*-value
**N° of anchors^b^**	16	(14‒18)	18	(16‒20)	< 0.001^2^
**N° of levels fused^a^**	11.5	(1.6)	11.3	(1.6)	0.521^1^
**Duration of surgery (min)^b^**	202	(179‒219)	190	(167‒210)	0.008^2^
**Length of stay (days)^b^**	6	(5‒7)	6	(5‒7)	0.539^2^

Values are presented as the ^a^ mean (SD), ^b^ median (Q1‒Q3), or number (%).

^1^ t-test; ^2^ Mann-Whitney test.

**Table 3:** Blood loss parameters and blood transfusion of adolescents who underwent surgery for idiopathic scoliosis with or without Tranexamic Acid (TXA).

		Non TXA (n = 116)	TXA (n = 71)	*p*-value
**EBL/EBV × 100 (%)**		38.5 [24.0–57.1]	24.3 [15.9–37.0]	< 0.001^a^
**EBL (mL)**		1437.5 [957.5–2025.5]	900.0 [600.0–1313.0]	< 0.002^a^
**Transfusion PRBC intraoperative (n° patients)**	No	50 (43.1%)	59 (83.1%)	< 0.001^b^
	Yes	66 (56.9%)	12 (16.9%)	
**Transfusion PRBC postoperative (n° patients)**	No	52 (44.8%)	42 (59.2%)	0.057^b^
	Yes	64 (55.2%)	29 (40.8%)	
**Cumulative transfusion (n° patients)**	No	24 (20.7%)	33 (46.5%)	< 0.001^b^
	Yes	92 (79.3%)	38 (53.5%)	

Values are expressed as medians [interquartile range] for continuous variables and numbers (percentages) for categorical variables. Statistical test: ^a^Wilcoxon rank-sum test; ^b^(Mann-Whitney); χ² test. EBL, Estimated Blood Loss; EBV, Estimated Blood Volume; PRBC, Packed Red Blood Cell.

In the multivariable linear regression model (Table 4), TXA use was associated with a lower percentage of estimated blood volume loss (β = -12.7, 95% CI -21.0 to -4.4; *p* = 0.003). Greater body weight was associated with increased blood loss (*p* < 0.001). Male sex was also associated with greater blood loss (*p* = 0.021) (Table 4). Operative factors showed strong associations with blood loss: each additional fused level was associated with a 4.8% increase in estimated blood volume loss (β = 4.8, 95% CI 2.0 to 7.6; *p* = 0.001), and longer surgical duration was associated with higher blood loss (β = 0.2, 95% CI 0.1 to 0.3; *p* = 0.004). Age (*p* = 0.97) and Cobb angle (*p* = 0.27) were not significantly associated with blood loss. In the unadjusted analysis, TXA use was associated with lower perioperative blood loss expressed as percentage volemia loss (-17.4, 95% CI -24.7 to -10.1; *p* < 0.001). In the primary multivariable linear regression model adjusting for prespecified clinical and surgical covariates, TXA use remained negatively associated with percentage volemia loss (-12.7, 95% CI -19.7 to -5.8; *p* < 0.001) (Fig. S2‒S3 in the Supplementary Material).

**Table 4:** Univariable and multivariable regression analysis of factors associated with percentage estimated blood loss volume (EBL/EBV × 100).

Variable	Univariable analysis			Multivariable analysis		
	Coefficient	(95% CI)	*p*-value	Coefficient	(95% CI)	*p-*value
TXA						
Non TXA	Reference	‒	‒	Reference	‒	‒
TXA	−17.4	(−24.7; -10.1)	< 0.001	−12.7	(−21.0; -4.4)	0.003
Age (years)	−1.7	(−4.0; 0.6)	0.140	0.0	(−2.3; 2.3)	0.972
Weight (kg)	−0.7	(−1.0; -0.4)	< 0.001	−0.8	(−1.1; -0.4)	< 0.001
Gender						
Female	Reference	‒	‒	Reference	‒	‒
Male	4.8	(−10.6; 20.3)	0.538	15.0	(2.3; 27.7)	0.021
ASA						
ASA I	Reference	‒	‒	Reference	‒	‒
ASA II	−8.3	(−16.5; -0.1)	0.049	−9.1	(−16.3; -1.8)	0.015
ASA III	−8.3	(−24.9; 8.3)	0.327	−25.0	(−48.1; -1.9)	0.034
Cobb angle (degrees)	0.4	(0.1; 0.6)	0.008	0.2	(−0.1; 0.4)	0.269
Number of fused levels	4.7	(1.8; 7.5)	0.001	4.8	(2.0; 7.6)	0.001
Number of screws	−0.5	(−1.8; 0.8)	0.469	−1.3	(−2.8; 0.1)	0.076
Surgery duration (min)	0.2	(0.1; 0.4)	< 0.001	0.2	(0.1; 0.3)	0.004
Type of curve						
Dorsal	Reference	‒	‒	Reference	‒	‒
Lumbar	−11.8	(−20.4; -3.2)	0.007	−13.6	(−24.9; -2.2)	0.019
Dorsolumbar	−7.9	(−17.3; 1.5)	0.097	−6.5	(−16.4; 3.5)	0.200

Values of the regression are from linear models fitted with robust standard errors. The multivariable model includes Tranexamic Acid (TXA), age, weight, gender, ASA status, Cobb angle, number of fused levels, number of screws, duration of surgery, and scoliosis type.

Graphical assessment of residuals demonstrated mild deviations from normality and non-constant variance across fitted values. The Breusch-Pagan/Cook-Weisberg test provided evidence of heteroscedasticity (χ² = 101, *p* < 0.001). Accordingly, all models are reported using heteroscedasticity-consistent (robust) standard errors (Fig. S8 in the Supplementary Material). Coefficient estimates were unchanged compared with conventional models, but robust variance estimation was considered more appropriate given the observed error structure.

Given baseline imbalances in the number of fused levels and number of pedicle screws, the three sensitivity models fitted, the association between TXA use and percentage volemia loss remained consistently negative with overlapping confidence intervals, supporting the robustness of the primary findings (Fig. S4‒S6 in the Supplementary Material).

Propensity scores for TXA use were estimated based on clinically relevant variables. Kernel density plots demonstrated adequate overlap between TXA and non-TXA groups (Fig. S9 in the Supplementary Material). The stabilized inverse probability of IPTW was then applied. In the marginal IPTW model including TXA only, TXA use remained associated with a lower percentage volemia loss (-18.5, 95% CI -33.8 to -3.2; *p* = 0.018). (Fig. S10 in the Supplementary Material) In the doubly robust IPTW model, additionally adjusting for the same covariates as the primary analysis, the association persisted with a similar direction and magnitude (-13.4, 95% CI -19.9 to -6.8; *p* < 0.001). Weight distributions are shown in Fig. S10 in the Supplementary Material. Together, these analyses indicate that the association between TXA use and lower percentage volemia loss is robust across regression-based and propensity-score-weighted approaches (Fig. S9‒S12 in the Supplementary Material).

## Discussion

This study is the first that we are aware of to provide evidence that a low dose regimen for TXA for AIS 39% reduction in blood loss and in PRBC transfused. The use of a homogeneous patient population and a consistent team of surgeons and anesthesiologists helped reduce clinical and procedural variability, thereby limiting potential confounding in this retrospective observational cohort study evaluating the association between tranexamic acid use and clinical outcomes. When the patient cohort is uniform in terms of baseline characteristics (such as age, comorbidities, and surgical indication), and the surgical and anesthetic management is standardized by the same team, the likelihood that differences in outcomes are due to factors other than the intervention (low dose of TXA) is substantially reduced. Previous studies have aimed to identify predictive factors for blood loss in AIS surgery. In our cohort study, we investigated the factors influencing blood loss in surgical patients and found significant correlations with the use of TXA, ASA physical status, lumbar curve, the number of levels fused, and operation duration. After adjustment for patient and surgical factors, TXA was associated with a 12.7% absolute reduction in estimated blood volume loss compared with no TXA. These findings contribute to the growing body of evidence supporting the efficacy of TXA in reducing the percentage of intraoperative estimated blood loss and highlight the importance of considering multiple variables in managing surgical blood loss.

Halpern et al. found that TXA, along with sex, surgical duration, and major coronal curve, was associated with blood loss in PSF, explaining 24% of the variation.^16^ Hasan et al. observed that surgical blood loss increased with male sex, each vertebral level fused, and additional surgical time.^17^ Goobie et al., using a dose of 50 mg.kg^-1^ bolus and 10 mg.kg^-1^.h^-1^ infusion, found that TXA, duration of surgery, and the number of spinal levels fused were significant predictors of blood loss, with TXA reducing blood loss by 223 mL.^18^ Four studies using very high doses of TXA (100 mg.kg^-1^ loading and 10 mg.kg^-1^.h^-1^ maintenance) showed significant reductions in EBL and transfusion requirements in idiopathic and neuromuscular scoliosis patients. Xu's study with a 20 mg.kg^-1^ loading dose and 10 mg.kg^-1^.h^-1^ maintenance dose demonstrated significant decreases in EBL and transfusion needs.^19^, ^20^, ^21^ Lastly, two authors reported that a low-dose TXA regimen (10 mg.kg^-1^ loading, 1 mg.kg^-1^.h^-1^ maintenance) significantly decreased total blood loss and blood product transfusion, though not specifically PRBCs.^22^^,^^23^

Since TXA demonstrates a ceiling effect, and side effects are dose-dependent, dose optimization is essential. High doses, especially in cardiac surgery, increase seizure risk. Lecker et al. emphasized dose-dependent neurotoxicit.^24^ Balancing efficacy and safety is therefore critical. Before TXA, transfusion rates ranged from 36%–75% in spinal surgery. In our study, low-dose TXA reduced both blood loss and transfusion requirements during AIS surgery.

TXA has been used in different combinations of loading dose and maintenance doses, varied by a factor of 10, with loading doses ranging from 2–100 mg.kg^-1^, associated with a 2–100 mg.kg^-1^ continuous infusion. We found that there is a large variability in dose schemes observed among the different studies, with no rationale for using certain specific doses. The doses used in those trials are not based on pharmacokinetics studies, which have not been assessed for this specific population and setting. However, pharmacokinetic evidence suggests the use of a 10–15 mg.kg^-1^ loading dose, followed by an infusion of 1 mg.kg^-1^.h^-1^ or a repeated dose. The same dose is also recommended in the ESAIC 2023^25^. Initiating a low-dose TXA protocol for routine use is scientifically supported by evidence demonstrating that low-dose regimens are effective in reducing perioperative blood loss and transfusion requirements, while maintaining a favorable safety profile.^26^, ^27^, ^28^, ^29^, ^30^ Therefore, we decided to use a 10 mg.kg^-1^ bolus for 15 minutes, plus 1 mg.kg^-1^.h^-1^ from incision up to surgical wound closure after surgery. The same was done by Neilipovitz et al. where they prospectively randomized children scheduled for scoliosis surgery to receive 10 mg.kg^-1^ TXA followed by a continuous infusion of 1 mg.mL^-1^.h^-1^ or the same infusion scheme with saline. The authors reported that 28% less blood was given in the TXA group compared with the placebo.^23^ Verma et al. also demonstrated that lower doses of TXA reduced blood loss but not transfusion rate.^22^ On the other hand, Sethna et al. and Lykissas et al. demonstrated that using high doses of TXA at 100 mg.kg^-1^ load followed by 10 mg.kg^-1^.h^-1^ significantly decreased the total blood loss when compared with placebo; however, the transfusion requirement did not significantly decrease.^19^, ^20^, ^21^ Grant et al. compared two doses of TXA, demonstrated that higher-dose TXA is likely effective at reducing perioperative transfusion requirements for children undergoing posterior instrumentations and fusion surgery for idiopathic scoliosis (50%).^31^ Although only adolescent idiopathic scoliosis patients were examined, the number of patients included in the study was too low, and the surgeries were performed by different surgeons. In our cohort study, we have always had the same team of orthopedic surgeons, and 116 patients received TXA compared to the control group (71 patients non-TXA). Meanwhile, Johnson et al. described that high-dose TXA is more effective than low-dose TXA in reducing blood loss and transfusion requirements in pediatric idiopathic scoliosis patients undergoing surgery.^32^ Burney et al. described that since the introduction of TXA, no patient has required intraoperative or postoperative allogeneic blood product transfusion.^33^ The main issue is that all the studies were performed with different doses of bolus and perfusion of TXA. Despite varied regimens, all studies show patient benefit; in our series, blood volume loss fell by approximately 39% with low-dose TXA.

Furthermore, considering the etiology of the disorder, secondary scoliosis, neuromuscular, or other congenital conditions are associated with substantial intraoperative blood loss compared to AIS. Factors implicated in the main blood loss include seizure medication, poor nutritional status, depletion of clotting factors, and abnormality in platelet aggregation.^20^ Shapiro et al. found a 46% decrease in blood loss and a 46% decrease in transfusion volumes in a retrospective study examining blood loss in spinal fusions secondary to Duchenne muscular dystrophy.^20^ Antifibrinolytics were effective in decreasing intraoperative blood loss during posterior spinal fusion and instrumentation in children with cerebral palsy; in fact, TXA was found to be more efficacious than EACA.

The Cochrane database review of antifibrinolytic agents for reducing blood loss in scoliosis surgery in children stated that antifibrinolytics reduce blood loss and blood transfusion in scoliosis surgery. However, the fact that only six studies met their inclusion criteria and no comment on dosing regimens was made shows that there is scope for further research in this area.^34^^,^^35^

The major concern surrounding the use of TXA is the potential increased risk of thromboembolic events. Thromboembolic complications after spinal fusion are a relatively rare event in children. The risk of venous thromboembolic events in children who undergo spinal fusion with a diagnosis of AIS was found to be approximately 0.04%.^36^ TXA does not alter blood clotting, but rather slows the dissolution of the blood clots. Reports of dose-dependent side effects from TXA on clinical seizures mandate the lowest effective dose possible to maximize efficacy while limiting adverse events. All patients were evaluated during a routine orthopedic follow-up visit two weeks after hospital discharge, and no complications were reported; however, because no standardized protocol for systematic adverse-event screening was in place and safety assessment relied on routine clinical practice in this retrospective setting, some complications, particularly subclinical or delayed events, may have been under-detected. However, the study lacked sufficient statistical power to support any conclusions regarding safety. While some predictive factors, such as gender and the number of levels fused, are not modifiable, male sex emerged as an independent predictor of greater blood loss in our cohort. This finding is consistent with the results of Lalenti et al,^4^ who also identified male sex as a significant predictor of increased blood loss in posterior spinal fusion procedures. The concordance between these results supports the hypothesis that sex-related anatomical or physiological differences, such as greater muscle mass and soft tissue dissection in male patients, may contribute to increased intraoperative bleeding.^4^ Although the exact mechanisms remain speculative, our findings reinforce the relevance of sex as a clinically meaningful factor when estimating perioperative blood loss risk.

Similar to previous studies, our study supports the findings that blood loss increased with fused vertebrae and longer operative time.^4^^,^^37^ The number of fused levels showed a strong dose-response relationship with bleeding, with each additional level associated with an average 4.8% increase in estimated blood volume loss. Surgical duration was also an important determinant, with each additional unit of operative time associated with a 0.2% increase in blood volume lost. These findings are clinically intuitive and highlight that procedural complexity and operative exposure substantially influence blood loss risk, reinforcing the need to account for these factors when evaluating the effect of antifibrinolytic therapy. Thompson et al. identified factors correlated with massive hematic losses; the number of instrumented levels was the variable with the greatest impact, especially in surgeries with 12 instrumented levels. Yu et al. correlated low weight, a Cobb angle above 50%, more than 6 instrumented levels, and the introduction of osteotomy with massive loss (> 30% of blood volume) that occurred in 59.9% of their patients.^38^ However, our study did not find a correlation between blood loss and Cobb angle.

Our study has some limitations. The main limitation of this study is its retrospective design; because treatment was not randomized, differences in baseline characteristics and clinical decision-making may have influenced both TXA administration and blood loss. In our study, the difference in the number of patients between the two groups can be attributed to the temporal aspect of data collection. As data were collected over a period, variations in patient inclusion naturally occurred. To address group imbalance and potential confounding, we applied multivariable regression with heteroscedasticity-consistent standard errors and conducted prespecified sensitivity analyses based on alternative model specifications and propensity score weighting. The consistency of results across these approaches supports the robustness of the primary findings. By doing so, we aimed to balance the groups and mitigate the impact of any confounding variables, ensuring a more accurate and unbiased association estimates in our analysis.

Other limitation was the number of TXA-treated patients who received a transfusion was small, limiting statistical power and increasing uncertainty around transfusion-related estimates. Residual confounding remains possible despite prespecified covariate adjustment and multiple sensitivity analyses. Several surgically relevant variables were imbalanced at baseline, and although alternative model specifications and propensity score-weighted analyses yielded consistent results, unmeasured confounding cannot be excluded. The use of complete-case analysis may also have introduced selection bias, and some linear model assumptions were only partially satisfied despite the application of robust variance estimators. Another limitation is that the indications for transfusion were not defined with objective parameters. The restrictive policy may be one of the most important measures missed in this study. Lacroix et al showed that a restrictive policy with a threshold below 7 g.dL^-1^ applied to children hospitalized in intensive care units significantly reduced transfusions with no increase in morbidity and mortality.^39^ Anesthesiologists, however, followed common practice triggers such as an intraoperative hemoglobin level less than 8 g.dL^-1^. Our study reflects real-world practice, where transfusion is often guided by clinical assessment and general recommendations rather than rigid criteria, introducing variability in transfusion rates and potentially confounding the evaluation of tranexamic acid efficacy. For example, the study by Sui et al. retrospectively reviewed adolescent idiopathic scoliosis patients and reported transfusion requirements and blood loss, but did not specify a strict transfusion protocol; instead, transfusion was administered according to clinical indications, which typically follow international guidelines but allow for provider discretion.^40^ Similarly, Johnson et al. conducted a retrospective cohort study comparing high-dose and low-dose tranexamic acid in pediatric scoliosis surgery, with transfusion decisions made by the clinical team rather than by a fixed hemoglobin threshold.^32^ Blood loss calculations were estimated by a uniform method.

## Conclusion

This study indicates that low-dose TXA use was associated with a lower percentage of intraoperative estimated blood volume loss and transfusion requirements, with no observed increase in thrombotic events. Independent predictors of blood loss included TXA use, surgery duration, and the number of fused spinal levels. These findings highlight the role of tranexamic acid in blood loss management while underscoring the need for further research to develop predictive models and improve outcomes in scoliosis surgery. Future prospective, randomized controlled trials are warranted to confirm these results and to further define the role of low-dose tranexamic acid within patient blood management strategies for adolescent idiopathic scoliosis surgery.

## Ethics

The study was approved by the Ethical Research Committee on 2015/6/5 of Hospital de Santo António, Centro Hospitalar Universitário de Santo António, Unidade Local de Saúde de Santo António (Santo António), Porto, Portugal, with the registration number 2015.083 (077-DEFI/072-CES).

## AI assistance disclosure

The authors confirm that no artificial intelligence tools were used in the preparation, writing, analysis, or editing of this manuscript.

## Data availability statement

The datasets generated and/or analyzed during the current study are available from the corresponding author upon reasonable request.

## Authors’ contributions

Paula Alexandra Sá: Conceptualization; methodology; formal analysis; investigation; resources; data curation; writing-original draft; writing-review & editing; visualization; project administration; funding acquisition.

Filipa Pereira: Conceptualization; methodology; investigation; data curation; writing-review & editing.

Daniel Soares: Conceptualization; methodology; investigation; data curation; writing-review & editing.

António Oliveira: Conceptualization; methodology; validation; writing-review & editing; supervision.

Eugénia Cruz: Conceptualization; methodology; validation; writing-review & editing; supervision.

Sibylle Langenecker: Conceptualization; methodology; validation; writing-review & editing; supervision.

All authors approved the final manuscript.

## Funding

This research did not receive any specific grant from funding agencies in the public, commercial, or not-for-profit sectors.

## Declaration of competing interest

The authors declare no conflicts of interest.

## Appendix Supplementary materials

Image, application 1

## Footnotes

Supplementary material associated with this article can be found in the online version at doi:10.1016/j.bjane.2026.844746.
