# Supplementary material for: Bleeding management in adolescent idiopathic scoliosis: the role of low-dose tranexamic acid
Source: Braz J Anesthesiol. 2026 Mar 7;76(2):844746. doi: 10.1016/j.bjane.2026.844746 (PMC13133522; doi:10.1016/j.bjane.2026.844746)

Supplementary Material

Table S1 STROBE checklist for reporting this retrospective cohort study on tranexamic acid use in adolescent idiopathic scoliosis surgery. The checklist indicates where each item is addressed in the manuscript, in accordance with the EQUATOR Network reporting guidelines.

	Item Description	Location (or reason for not reporting)
**Title and abstract**		
1a. Indicate the study's design	Indicate the study's design with a commonly used term in the title or the abstract.	Level of evidence section
1b. Abstract	Provide in the abstract an informative and balanced summary of what was done and what was found.	Abstract section
**Introduction**		
2. Background / rationale	Explain the scientific background and rationale for the investigation being reported.	Introduction section
3. Objectives	State specific objectives, including any prespecified hypotheses.	Introduction section, last paragraph
**Methods**		
4. Study design	Present key elements of study design early in the paper.	Methods section, paragraph 1
5. Setting	Describe the setting, locations, and relevant dates, including periods of recruitment, exposure, follow-up, and data collection.	Methods section, paragraph 1
6a. Eligibility criteria	**Cohort study:** Give the eligibility criteria, and the sources and methods of selection of participants. Describe methods of follow-up. **Case-control study:** Give the eligibility criteria, and the sources and methods of case ascertainment and control selection. Give the rationale for the choice of cases and controls. **Cross-sectional study:** Give the eligibility criteria, and the sources and methods of selection of participants.	Methods section: Participations and procedures, paragraph 1
6b. Matching criteria	**Cohort study:** For matched studies, give matching criteria and number of exposed and unexposed. **Case-control study:** For matched studies, give matching criteria and the number of controls per case.	Results: Participants and procedures, paragraph 1
7. Variables	Clearly define all outcomes, exposures, predictors, potential confounders, and effect modifiers. Give diagnostic criteria, if applicable.	Outcomes, paragraph 1
8. Data sources / measurement	For each variable of interest give sources of data and details of methods of assessment (measurement). Describe comparability of assessment methods if there is more than one group.	Outcomes, paragraphs 2and 3
9. Bias	Describe any efforts to address potential sources of bias.	Outcome parameters, paragraph 1 and 2 and 3
10. Study size	Explain how the study size was arrived at.	Results

		Participants and procedures, paragraph 1<br>(all eligible patients in this period were included, and no formal sample size calculation was performed)
11. Quantitative variables	Explain how quantitative variables were handled in the analyses. If applicable, describe which groupings were chosen, and why.	Statistical analysis, paragraph 1
12a. Statistical methods	Describe all statistical methods, including those used to control for confounding.	Methods section, Statistical analysis subsection
12b. Statistical methods – subgroups and interactions	Describe any methods used to examine subgroups and interactions.	Methods section<br>Statistical analysis, paragraph 1
12c. Statistical methods – missing data	Explain how missing data were addressed.	Results<br>Participants and procedures, paragraph 1<br>(excluding patients with missing data)
12di. Statistical methods – loss to follow-up	**Cohort study:** If applicable, describe how loss to follow-up was addressed.	This was a retrospective cohort study using electronic medical records. Follow-up information was available for all included participants; therefore, loss to follow-up was not applicable
12dii. Statistical methods – matching cases and controls	**Case-control study:** If applicable, explain how matching of cases and controls was addressed.	Not applied
12diii. Statistical methods – sampling strategy	**Cross-sectional study:** If applicable, describe analytical methods taking account of sampling strategy.	Not applied
12e. Statistical methods – sensitivity analyses	Describe any sensitivity analyses.	Other robustness checks or quality controls were applied that the authors deemed sufficient
**Results**		
13a. Participant numbers	Report the numbers of individuals at each stage of the study—e.g., numbers potentially eligible, examined for eligibility, confirmed eligible, included in the study, completing follow-up, and analysed; Consider use of a flow diagram.	Results, paragraph 1
13b. Participants – non-participation	Give reasons for non-participation at each stage.	Results, paragraph 1
13c. Participants – flow diagram	Consider use of a flow diagram.	Not applied

14a. Descriptive data – participant characteristics	Give characteristics of study participants (e.g., demographic, clinical, social) and information on exposures and potential confounders. Present the information in a table.	Table 1 and table 2
14b. Descriptive data – missing data	Indicate the number of participants with missing data for each variable of interest.	Results<br>Participants and procedures, first phrase
14c. Descriptive data – follow-up time	**Cohort study:** Summarise follow-up time—e.g., average and total amount.	Results<br>Participants and procedures, last phrase
15. Outcome data	**Cohort study:** Report numbers of outcome events or summary measures over time. **Case-control study:** Report numbers in each exposure category, or summary measures of exposure. **Cross-sectional study:** Report numbers of outcome events or summary measures.	Table 3 and Table 4
16a. Main results	Give unadjusted estimates and, if applicable, confounder-adjusted estimates and their precision (e.g., 95% confidence intervals). Make clear which confounders were adjusted for and why they were included.	Results<br>Participants and procedures, last paragraph<br>Table 4
16b. Main results – category boundaries	Report category boundaries when continuous variables were categorised.	Continuous variables analyzed in their original form; no categorization was applied
16c. Main results – risk	If relevant, consider translating estimates of relative risk into absolute risk for a meaningful time period.	Table 4
17. Other analyses	Report other analyses done—e.g., analyses of subgroups and interactions, and sensitivity analyses.	Not applied
**Discussion**		
18. Key results	Summarise key results with reference to study objectives.	Results section
19. Limitations	Discuss limitations of the study, taking into account sources of potential bias or imprecision. Discuss both direction and magnitude of any potential bias.	Discussion section, last paragraph
20. Interpretation	Give a cautious overall interpretation considering objectives, limitations, multiplicity of analyses, results from similar studies, and other relevant evidence.	Discussion section
21. Generalisability	Discuss the generalisability (external validity) of the study results.	Conclusion section
**Other information**		
22. Funding	Give the source of funding and the role of the funders for the present study and, if applicable, for the original study on which the present article is based.	Funding section

Fig. S1 Flowchart of patients and exclusion criteria.

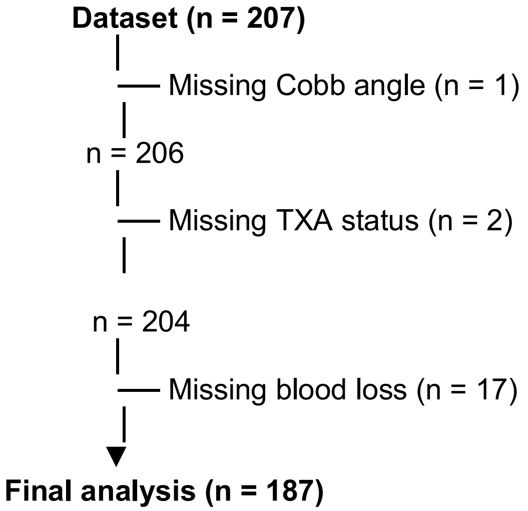

Table S2 Standardized mean differences (SMDs) for baseline comparing TXA and non-TXA.

Variable	Type	SMD (TXA vs non-TXA)
Age	continuous	0.067
Weight	continuous	0.005
Cobb angle	continuous	-0.171
Number of levels	continuous	-0.097
Number of screws	continuous	0.698
Duration of surgery	continuous	-0.386
Percentage volemia	continuous	-0.664
Gender	binary	0.132

SMD: Standardized mean differences.

Confounding and choice of adjustment variables

Fig. S2 Crude model: TXA only (unadjusted).

```
. regress percentage_volemia i.atx, vce(robust)

Linear regression                               Number of obs     =        187
                                                F(1, 185)         =      22.18
                                                Prob > F          =     0.0000
                                                R-squared         =     0.0852
                                                Root MSE          =     27.846
```

percentage~a	Coefficient	Robust std. err.	t	P>|t|	[95% conf.	interval]
atx						
TXA	-17.41745	3.698213	-4.71	0.000	-24.71354	-10.12135
_cons	45.99914	3.006286	15.30	0.000	40.06813	51.93015

Fig. S3 Primary adjusted model (Full): TXA + prespecified confounder set (age, sex, weight, ASA, Cobb angle, number of fused levels, number of screws, surgery duration, scoliosis type).

```
. regress percentage_volemia i.atx `adj_full', vce(robust)

Linear regression                               Number of obs     =        186
                                                F(12, 173)        =       5.04
                                                Prob > F          =     0.0000
                                                R-squared         =     0.3390
                                                Root MSE          =     24.383
```

percentage_v~a	Coefficient	Robust std. err.	t	P>|t|	[95% conf.	interval]
atx						
TXA	-12.7493	3.523369	-3.62	0.000	-19.70362	-5.794975
age	-.0454705	1.259331	-0.04	0.971	-2.531101	2.44016
weight	-.7657985	.1521815	-5.03	0.000	-1.06617	-.465427
gender						
male	15.02188	5.893721	2.55	0.012	3.389026	26.65474
asa						
2	-9.046547	3.599058	-2.51	0.013	-16.15026	-1.942829
3	-24.95219	10.09565	-2.47	0.014	-44.8787	-5.025683
cobbangle	.1503046	.1291195	1.16	0.246	-.1045477	.4051569
n_levels	4.754924	1.431015	3.32	0.001	1.930428	7.57942
nscrews	-1.312963	.7612634	-1.72	0.086	-2.815523	.1895971
dur_cirug	.1553342	.0462145	3.36	0.001	.0641174	.2465509
scoliosis_type						
lumbar	-13.65673	4.879568	-2.80	0.006	-23.28788	-4.02558
thoracolumbar	-6.467222	4.63727	-1.39	0.165	-15.62013	2.685689
_cons	20.49131	25.96819	0.79	0.431	-30.76396	71.74657

Fig. S4 Model without anchors: Excluding the number of screws while retaining the number of fused levels.

```
. regress percentage_volemia i.atx `adj_noScrews', vce(robust)

Linear regression                               Number of obs     =        186
                                                F(11, 174)        =       5.29
                                                Prob > F          =     0.0000
                                                R-squared         =     0.3268
                                                Root MSE          =     24.535
```

percentage_v~a	Coefficient	Robust std. err.	t	P>|t|	[95% conf.	interval]
atx						
TXA	-15.94857	3.317873	-4.81	0.000	-22.49703	-9.400113
age	.2809918	1.1873	0.24	0.813	-2.062372	2.624355
weight	-.7921045	.1536426	-5.16	0.000	-1.095348	-.4888615
gender						
male	15.56057	5.953815	2.61	0.010	3.809577	27.31156
asa						
2	-9.440847	3.608482	-2.62	0.010	-16.56288	-2.318818
3	-26.26954	10.66728	-2.46	0.015	-47.32345	-5.215622
cobbangle	.1420677	.1267429	1.12	0.264	-.1080837	.3922192
n_levels	3.388351	1.241063	2.73	0.007	.9388755	5.837826
dur_cirug	.1457069	.0478045	3.05	0.003	.0513556	.2400582
scoliosis_type						
lumbar	-14.60619	4.936407	-2.96	0.004	-24.34913	-4.863246
thoracolumbar	-7.754318	4.539883	-1.71	0.089	-16.71465	1.206011
_cons	14.6746	25.10671	0.58	0.560	-34.8783	64.2275

Fig. S5 Model without levels: Excluding number of fused levels while retaining number of screws.

```
. regress percentage_volemia i.atx `adj_noLevels', vce(robust)

Linear regression                               Number of obs     =        186
                                                F(11, 174)        =       5.00
                                                Prob > F          =     0.0000
                                                R-squared         =     0.2964
                                                Root MSE          =     25.084
```

percentage_v~a	Coefficient	Robust std. err.	t	P>|t|	[95% conf.	interval]
atx						
TXA	-15.53185	3.936269	-3.95	0.000	-23.30083	-7.762866
age	.2488445	1.238759	0.20	0.841	-2.196084	2.693773
weight	-.8048283	.1690481	-4.76	0.000	-1.138477	-.4711795
gender						
male	14.90875	6.642644	2.24	0.026	1.798224	28.01928
asa						
2	-9.431269	3.722761	-2.53	0.012	-16.77885	-2.083686
3	-22.08108	10.02863	-2.20	0.029	-41.8745	-2.287663
cobbangle	.1871587	.1353989	1.38	0.169	-.0800769	.4543942
nscrews	.0097965	.734857	0.01	0.989	-1.440585	1.460177
dur_cirug	.1887003	.0507779	3.72	0.000	.0884804	.2889201
scoliosis_type						
lumbar	-13.70411	5.1644	-2.65	0.009	-23.89704	-3.511182
thoracolumbar	-7.300355	4.74776	-1.54	0.126	-16.67097	2.070258
_cons	42.73616	25.99191	1.64	0.102	-8.563856	94.03618

Fig. S6 Model without anchors and levels: Excluding both number of fused levels and number of screws.

```
. regress percentage_volemia i.atx `adj_noLevNoScr', vce(robust)

Linear regression                               Number of obs     =        186
                                                F(10, 175)        =       5.38
                                                Prob > F          =     0.0000
                                                R-squared         =     0.2964
                                                Root MSE          =     25.012
```

percentage_v~a	Coefficient	Robust std. err.	t	P>|t|	[95% conf.	interval]
atx						
TXA	-15.50664	3.355348	-4.62	0.000	-22.1288	-8.884488
age	.2463042	1.159969	0.21	0.832	-2.043024	2.535633
weight	-.8046699	.1654736	-4.86	0.000	-1.131251	-.478089
gender						
male	14.90276	6.592433	2.26	0.025	1.891847	27.91366
asa						
2	-9.428289	3.690568	-2.55	0.011	-16.71204	-2.144538
3	-22.05858	9.744782	-2.26	0.025	-41.291	-2.826156
cobbangle	.1873564	.1353699	1.38	0.168	-.0798113	.4545241
dur_cirug	.1889021	.0511714	3.69	0.000	.0879095	.2898947
scoliosis_type						
lumbar	-13.69428	4.995471	-2.74	0.007	-23.55341	-3.83516
thoracolumbar	-7.289353	4.513391	-1.62	0.108	-16.19704	1.618331
_cons	42.8644	21.82413	1.96	0.051	-.2079883	85.93678

Diagnostics for the multivariable model

Fig. S7 Residuals analysis:

A) Residuals versus fitted values

B) Q-Q plot of residuals

C) P-P plot of standardized residuals

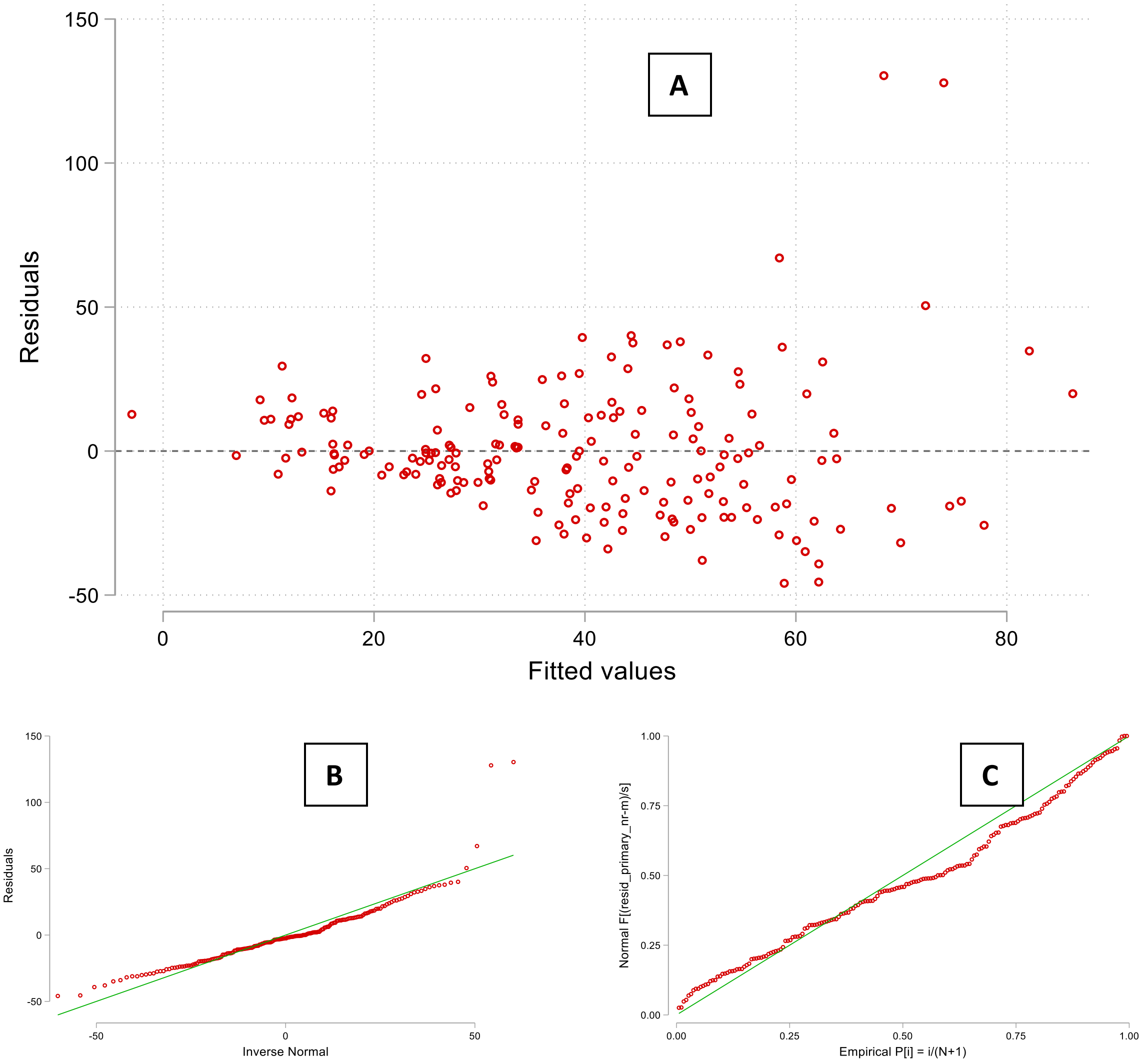

Fig. S8 Histogram of the residuals

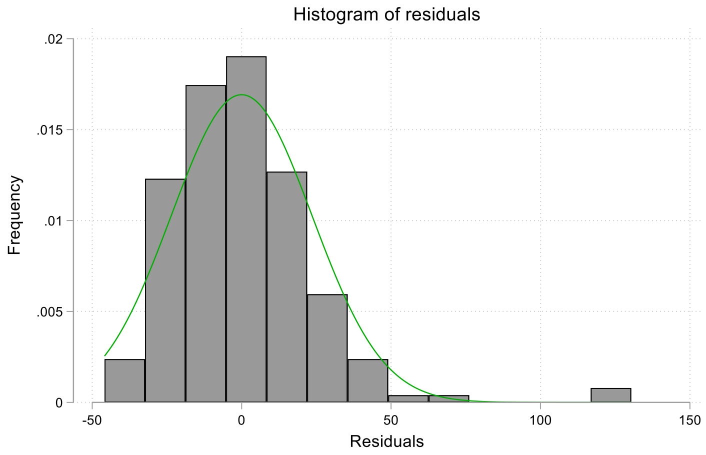

Propensity score sensitivity analysis

Fig. S9 Stata output of the marginal IPTW model (propensity score weights model without the additional covariates).

```
Linear regression                               Number of obs     =        187
                                                F(1, 185)         =       5.71
                                                Prob > F          =     0.0178
                                                R-squared         =     0.1018
                                                Root MSE          =     20.935

------------------------------------------------------------------------------
             |               Robust
percentage~a | Coefficient  std. err.      t    P>|t|     [95% conf. interval]
-------------+----------------------------------------------------------------
         atx |
        TXA  |  -18.53875   7.756046    -2.39   0.018    -33.84042   -3.237085
       _cons |   36.89061    4.38822     8.41   0.000     28.23322    45.54799
------------------------------------------------------------------------------
```

Fig. S10 Stata output of the marginal IPTW model (propensity score weights model without the additional covariates).

```
Linear regression                               Number of obs     =        186
                                                F(12, 173)        =       7.80
                                                Prob > F          =     0.0000
                                                R-squared         =     0.4061
                                                Root MSE          =     16.883
```

percentage_v~a	Coefficient	Robust std. err.	t	P>|t|	[95% conf.	interval]
atx						
TXA	-13.38146	3.327531	-4.02	0.000	-19.94925	-6.813675
age	-.3542336	1.526191	-0.23	0.817	-3.366585	2.658118
weight	-.7576766	.2299167	-3.30	0.001	-1.21148	-.3038737
gender						
male	11.33233	6.95247	1.63	0.105	-2.390255	25.05492
asa						
2	-12.37162	3.792375	-3.26	0.001	-19.8569	-4.886336
3	-34.13103	8.593407	-3.97	0.000	-51.09245	-17.16961
cobbangle	.3461027	.1840166	1.88	0.062	-.017104	.7093095
n_levels	5.033077	2.048279	2.46	0.015	.9902439	9.075911
nscrews	-2.046603	.578479	-3.54	0.001	-3.188389	-.9048182
dur_cirug	.1394063	.090341	1.54	0.125	-.0389061	.3177187
scoliosis_type						
lumbar	-8.425688	5.223013	-1.61	0.109	-18.73472	1.883345
thoracolumbar	11.56161	8.343855	1.39	0.168	-4.907248	28.03047
_cons	26.10568	31.5662	0.83	0.409	-36.19878	88.41014

Fig. S11 Propensity score overlaps by TXA status. Kernel density estimates of the propensity score for the TXA and non-TXA groups. Although the TXA group shows higher scores on average, there is reasonable overlap between groups, allowing propensity score weighting to be used for adjustment.

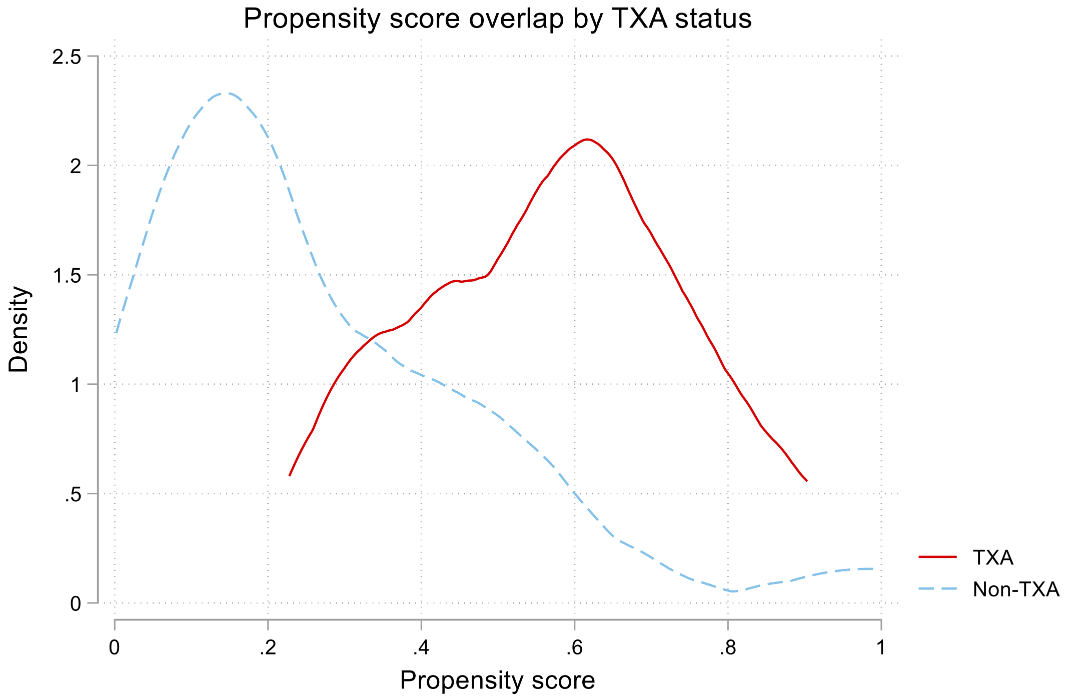

Fig. S12 Distribution of stabilized inverse probability of treatment weights (IPTW) before trimming. Most weights clustered near 1, with a small number of extreme values consistent with patients having very high or very low propensity for TXA.

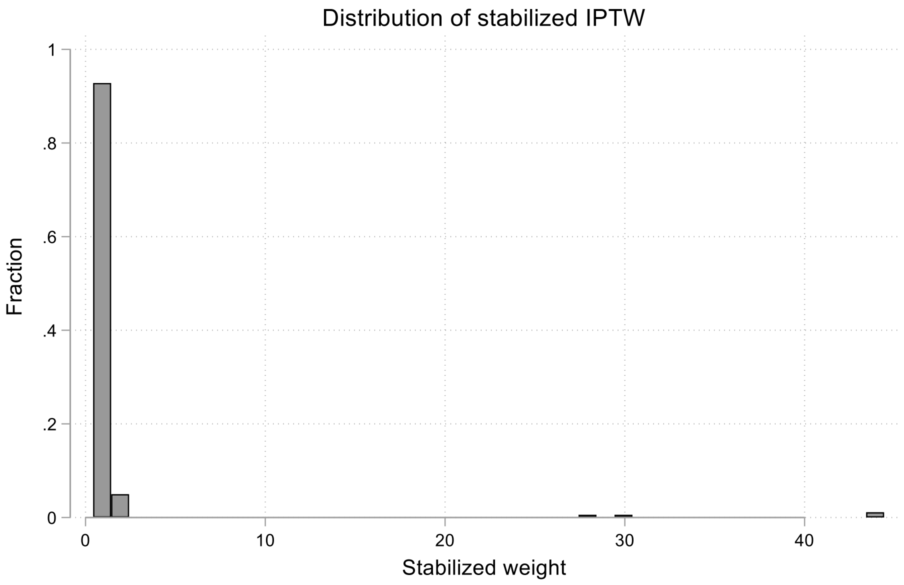

Supplement: Supplementary file 1 [file mmc1.pdf]
